# Restoration of Cyclo-Gly-Pro-induced salivary hyposecretion and submandibular composition by naloxone in mice

**DOI:** 10.1371/journal.pone.0229761

**Published:** 2020-03-10

**Authors:** Igor Santana Melo, Návylla Candeia-Medeiros, Jamylle Nunes Souza Ferro, Polliane Maria Cavalcante-Araújo, Tales Lyra Oliveira, Cassio Eráclito Alves Santos, Leia Cardoso-Sousa, Emilia Maria Gomes Aguiar, Stephanie Wutke Oliveira, Olagide Wagner Castro, Renata Pereira Alves-Balvedi, Luciano Pereira Rodrigues, Jandir M. Hickmann, Douglas Alexsander Alves, Igor Andrade Santos, Ana Carolina Gomes Jardim, Walter Luiz Siqueira, Angelo Ricardo Fávaro Pipi, Luiz Ricardo Goulart, Emiliano de Oliveira Barreto, Robinson Sabino-Silva

**Affiliations:** 1 Institute of Biological Sciences and Health, Federal University of Alagoas, Maceio, Alagoas, Brazil; 2 Optics and Materials Group, Optma, Federal University of Alagoas, Maceio, Alagoas, Brazil; 3 Institute of Biomedical Sciences, Federal University of Uberlandia, Uberlandia, Minas Gerais, Brazil; 4 College of Dentistry, University of Saskatchewan, Saskatoon, Saskatchewan, Canada; 5 Institute of Genetics and Biochemistry, Federal University of Uberlandia, Uberlandia, Minas Gerais, Brazil; 6 Institute of Engineering, Science and Technology, Federal University of Jequitinhonha and Mucuri’s Valley, Janaúba, Minas Gerais, Brazil; 7 National Centre for Sensor Research, School of Chemical Sciences, Dublin City University, Dublin, Ireland; 8 Department of Medical Microbiology and Immunology, University of California Davis, California, LA, United States of America; University of Arizona College of Medicine, UNITED STATES

## Abstract

Cyclo-Gly-Pro (CGP) attenuates nociception, however its effects on salivary glands remain unclear. In this study, we investigated the acute effects of CGP on salivary flow and composition, and on the submandibular gland composition, compared with morphine. Besides, we characterized the effects of naloxone (a non-selective opioid receptor antagonist) on CGP- and morphine-induced salivary and glandular alterations in mice. After that, in silico analyses were performed to predict the interaction between CGP and opioid receptors. Morphine and CGP significantly reduced salivary flow and total protein concentration of saliva and naloxone restored them to the physiological levels. Morphine and CGP also reduced several infrared vibrational modes (Amide I, 1687-1594cm^-1^; Amide II, 1594-1494cm^-1^; CH_2_/CH_3_, 1488-1433cm^-1^; C = O, 1432-1365cm^-1^; PO_2_ asymmetric, 1290-1185cm^-1^; PO_2_ symmetric, 1135-999cm^-1^) and naloxone reverted these alterations. The *in silico* docking analysis demonstrated the interaction of polar contacts between the CGP and opioid receptor Cys219 residue. Altogether, we showed that salivary hypofunction and glandular changes elicited by CGP may occur through opioid receptor suggesting that the blockage of opioid receptors in superior cervical and submandibular ganglions may be a possible strategy to restore salivary secretion while maintaining antinociceptive action due its effects on the central nervous system.

## Introduction

Saliva exerts multiple functions in the oral cavity such as protection against microorganisms, contribution to the taste and digestion and maintenance of oral health [1–3]. Salivary function is controlled by sympathetic and parasympathetic nervous system, which innervate acinar, ductal, myoepithelial and vascular cells in salivary glands [4,5]. The activation of muscarinic receptors in the acinar cells is the most important control of salivary flow rates [6]. Electrical stimulation of sympathetic efferent branch to the salivary glands results in a low flow of saliva which is rich in proteins [7]. Paradoxically, sympathectomy also generates decrease in salivary flow [8]. These findings demonstrate the complexity of the sympathetic regulation on salivary flow and salivary composition [9]. The activation of central pathways develops a great part in salivatory effects of intraperitoneal pilocarpine in rats [10].

Several substances with pharmacological properties can promote changes in salivary function. Morphine is an opioid receptor agonist that plays intense and long-lasting analgesia [11,12]. It was demonstrated that morphine increase lactate levels in serum, however its effects on salivary lactate concentration are unknown [13]. In humans and rats, the morphine administration was correlated to hyposalivation, and associated with changes in the ionic composition [14–15]. Bearing in mind that amylase is the most abundant protein in saliva [16], salivary amylase concentration decreased after morphine treatment [17]. It has been clearly demonstrated that morphine promotes reduction in the sympathetic activity to salivary glands by its action on the superior cervical ganglion and by inhibiting the release of neurotransmitter from postganglionic nerve endings [17]. Additionally, kappa-, delta-, and mu-opioid-receptor agonists are able to inhibit L-, N- and P/Q-types of calcium channels in submandibular ganglion neurons, indicating a reduction in parasympathetic activity to salivary glands [18]. The reduction on the parasympathetic nerve-induced salivary secretion generated by the morphine was partially reversed by naloxone, a non-selective opioid receptor antagonist. However, salivary secretion stimulated by intravenous infusion of acetylcholine was not reduced by morphine [19].

Cyclic dipeptides are among the smallest peptide derivatives frequently found in nature [20]. Cyclo-Gly-Pro (CGP) is an endogenous diketopiperazine derived from N-terminal tripeptide, glycine-proline-glutamate which is naturally cleaved from the insulin-like growth factor 1 (IGF-1) [21,22]. Previous studies have shown that CGP induces neuroprotective effects after ischemic brain injury [22]. CGP 35348 has an adjuvant role to produce a dose-dependent antagonism of antinociception [23]. Recently, our group demonstrated that the antinociceptive effect of CGP seemed to be mediated by the interaction with the opioid system, also reducing the hyper nociception and paw inflammation induced by carrageenan [24]. This might indicate the potential of CGP as a candidate for antinociceptive role with fewer side effects on salivary glands. It is important to emphasize that several effects of CGP in oral territories remain unknown.

Despite the knowledge about the effect of pharmacological agents on salivary glands, and consequently on salivary secretion, the CGP capacity to modulate submandibular and salivary components has never been investigated. Besides, it is important to highlight that the interaction between CGP and opioid receptors has also not been demonstrated. Thus, the aims of the present study were to investigate the CGP acute effects on salivary flow and composition, and on submandibular gland composition compared with morphine. Besides, we characterized the naloxone (a non-selective opioid receptor antagonist) effect on CGP- and morphine-induced salivary and glandular alterations in mice. After that, *in silico* analyses were performed to predict the 3D-interaction between the CGP and opioid receptors.

## Materials and methods

This study was carried out in strict accordance with the recommendations in the Guide for the Care and Use of Laboratory Animals of the Brazilian Society of Laboratory Animals Science (SBCAL). Experimental procedures were approved by the Ethical Committee of the Federal University of Alagoas (UFAL) (License 065/2011), according to Ethical Principles adopted by the Brazilian College of Animal Experimentation (COBEA). Animal studies are reported in compliance with the approved guidelines. To minimize the number of animals used and their suffering all effort were taken. Male Swiss mice (*Mus musculus*, 2 months) weighing 25–36 g were obtained from the breeding colonies of the UFAL and maintained at the Institute of Biological Sciences and Health rodent housing facility. Mice were randomly assigned to standard cages with five animals per cage. Animals were allowed free access to water and standard rodent chow diet and kept at 22 ± 2°C with a 12 h light/dark cycle, light on at 07:00h. To minimize circadian effects, all experimental procedures were conducted during the light phase. Power analysis was used as a basis to set the number of animals per experiment [25]. The number of samples was insert in each legend.

### Materials

All used reagents were of analytical grade and used without further purification. The following reagents were used: cyclo-Gly-Pro (CGP, ≥ 98% purity; Catalog number: 3705-27-9), morphine solution, naloxone and phosphate-buffered saline (Sigma Chemical Co., St. Louis, MO, USA).

### Experimental procedures

**Fig 1:**
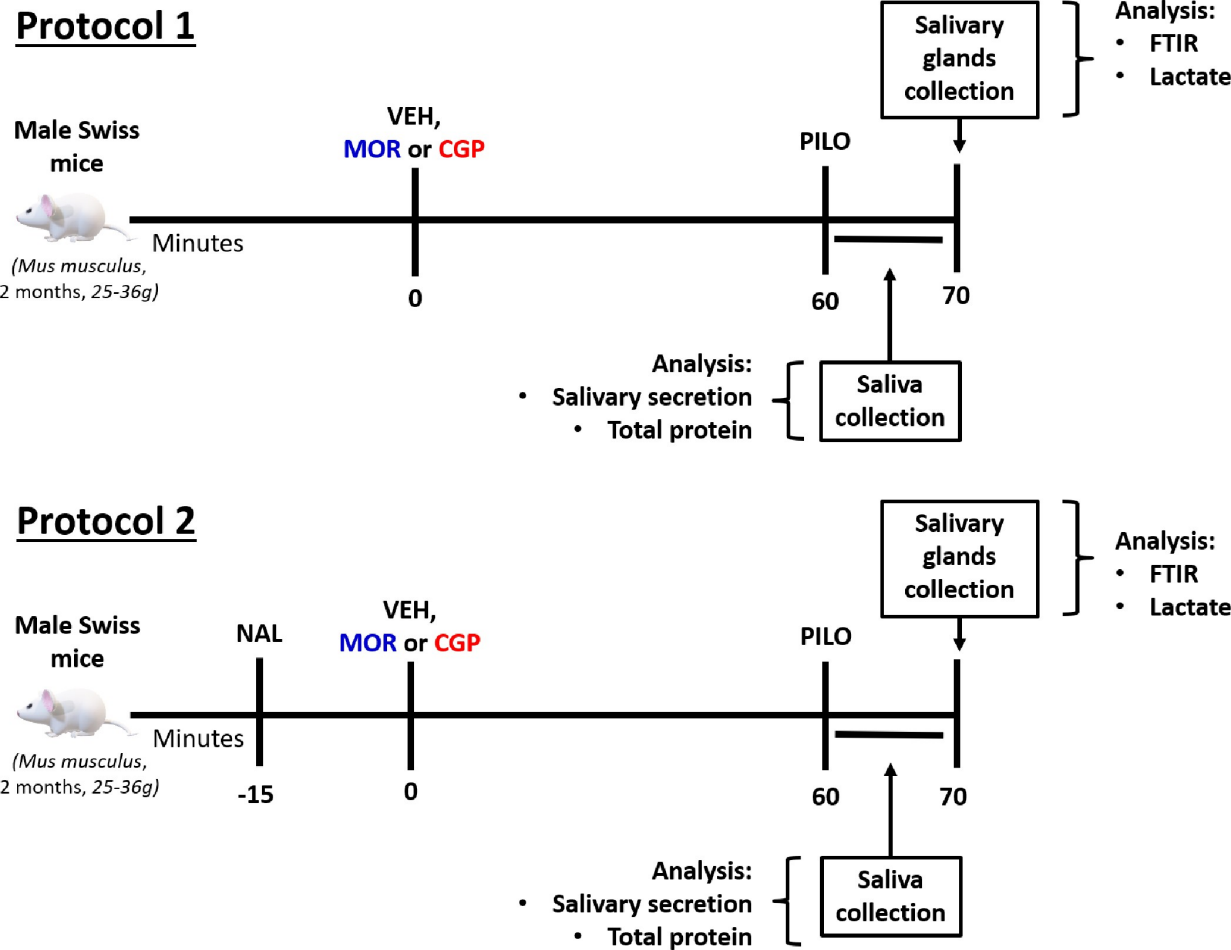
Experimental design of the acute treatment with vehicle, morphine or CGP in male Swiss mice in two protocols with or without the previous administration of naloxone. In protocol 1, mice were treated acutely with vehicle (VEH, NaCl, 0.9%), morphine (MOR, 17.5 μmol kg^-1^, ip) or CGP (1 μmol kg^-1^, ip). After one hour of treatment, a parasympathetic stimulus with pilocarpine (PILO, 2 mg kg^-1^, ip) was performed during 10 min for salivary secretion and salivary composition analysis. Immediately after saliva collection, submandibular glands were removed and storage at −80°C. The CGP and morphine effects on submandibular gland molecular composition were observed by Attenuated total reflectance Fourier transform infrared (ATR-FTIR) spectroscopy and lactate level analysis. In protocol 2, naloxone (15.3 μmol kg^-1^, ip) was administered 15 minutes prior to acute treatment with vehicle, morphine and CGP in order to evaluate the opioid receptors involvement in saliva and submandibular gland.

Animals were treated with vehicle (NaCl, 0.9%), morphine or CGP (CGP, ≥ 98% purity) (Fig 1, Protocol 1). Morphine was applied at dose of 17.5 μmol kg^-1^ (0.1 ml/10g, i.p.) and CGP 1 μmol kg^-1^ (0.1 ml/10g, i.p.), considering the similar antinociceptive effects observed in the hot plate test [24]. For randomization, vehicle was injected in the control group, while the other mice received morphine or CGP. Blinding was implemented as follows: the operator was blinded to the group identity, but not to animals of the same group. Thus, for i.p. drug injection, different solutions were prepared: vehicle, morphine and CGP. In order to analyze the opioid receptors involvement in salivary secretion and changes in submandibular composition, similar analysis was performed in another set of animals under naloxone administration (pre-treatment)(15.3 μmol kg^-1^, i.p.), an opioid receptor antagonist, 15 minutes before treatment with vehicle, morphine or CGP (Fig 1, Protocol 2).

### Saliva and salivary glands collection

One hour after treatment with vehicle, morphine or CGP, the animals were intraperitoneally anesthetized (xylazine 5 mg kg^-1^body weight; ketamine 35 mg kg^-1^) and then parasympathetic stimulation was performed for salivary secretion through pilocarpine injection (2 mg kg^-1^, i.p.). Total saliva was collected for 10 min from the oral cavity [26]. After that, submandibular and parotid glands were collected and weighted [27]. Salivary secretion was calculated based on volume of saliva acquired in 10 minutes collection divided by the weight of the salivary gland tissues (μl/g tissue) (Fig 1).

### Total protein concentration of saliva

Total protein concentration was measured using Bradford Protein assay. Values were expressed in mg/ml using serum albumin as standard protein. [28].

### Saliva analysis by dispersive x-ray analysis system (EDX)

Pilocarpine-stimulated saliva from Protocol 1 animals was used to assess the inorganic elements by EDX (Fig 1). In each experiment,15 μL of saliva were used to measure the ionic composition. EDX mode was set up as 180 seconds per sample ion detection using Si (Li) semiconductor detector (Shimadzu, Fukuoka, Japan) at 30 kV in a vacuum chamber. Ions quantification was taken from the excitement of their electrons.

### Lactate concentration of submandibular glands

Submandibular gland specimens were tested using an enzymatic system for lactate quantitative determination (Labtest, Brazil). Experiment was done according to the manufacturer’s instruction. The submandibular tissue was removed, washed using physiological saline (NaCl 0.9%), and immediately frozen at—80°C. Frozen gland tissue was homogenized in a phosphate buffer (1 : 10 w/v, pH 7.4). To measure the lactate concentration, the homogenate (25 μg) was incubated in a solution with 4- aminoantipyrine (50 mmol/L), peroxidase, L-lactate oxidase (1,000 U/L) e N-ethyl-N-(2-hydroxy-3-sulfopropyl)-3-methylaniline (1.5 mmol/L) and sodium azide (0.09%) at 37°C for 5 minutes using a spectrophotometer at 340 nm.

### Histology

The histological analysis in submandibular glands were performed only in animals described in protocol 1 (Fig 1). Submandibular glands were fixed in 10% buffered formalin. Subsequently, these glands were dehydrated in alcohol (80, 90 and 100%), cleared in xylene and embedded in paraffin. Histological sections with a thickness of 5 μm were acquired using a microtome (Leica RM2125). Then these sections were placed on slides and stained with hematoxylin and eosin. Histological slides were examined and micrograph pictures were obtained using an optical microscope (Olympus BX41).

### Acetylcholinesterase activity of submandibular glands

The acetylcholinesterase (AChE) activity quantification in submandibular glands were performed only in animals described in protocol 1 (Fig 1) using a acetylcholine as substrate (Ach, Sigma Chemical, St. Louis, Mo, USA) at room temperature. The submandibular glands were homogenized in saline (1:10, 0.9%), through a tissue shredder (Polytron®). In a 96-well plate, acetylcholine (50μL) was added, and then color reagent was added and taken to incubator for 3 minutes at 37°C. Subsequently, sample (20μL) was added [the first well received deionized water (20μL) and the second the standard solution (20μL)] and then taken back to the water bath for 2.5 minutes at 37°C. Finally, a blocked solution (150μL) was added for subsequent reading at the spectrophotometer (at absorbance 410nm). The AChE activity was determined by the sample value product. Enzyme activity values were calculated after normalization by a total protein concentration measured using Bradford assay.

### Molecular profile in submandibular glands by ATR-FTIR spectroscopy

Submandibular glands spectra were recorded in 4000–400 cm-1 region using FTIR spectrophotometer Vertex 70 (Bruker Optik) using a micro-attenuated total reflectance (ATR) accessory. The fingerprint region was chosen to be displayed due to the interest region. All spectra were recorded at room temperature (23±1°C). The crystal material unit in ATR unit was a diamond disc as internal-reflection element. The sample penetration depth ranges between 0.1 and 2 μm and depends on the wavelength and the refractive index of the ATR-crystal material. In the ATR-crystal the infrared beam is reflected at the interface toward the sample. Twenty mg of submandibular were lyophilized using a rotary evaporator (Thermo Savant, San Jose, CA) to obtain sample spectra. The air spectrum was used as a background in ATR-FTIR analysis. Samples spectrum were taken with 4 cm^-1^ of resolution and 32 scans were performed to each analysis. The ATR-FTIR spectra were also baseline corrected using OPUS software [29]. Table 1 shows the frequencies and assignments of the vibrational modes identified in submandibular glands. Briefly, the vibrational mode between 1687–1594 cm^-1^ is identified as υ_N_H (Amide I) bending vibrations [29–31]. The δ_N_H (Amide II) bending vibration is usually represented between 1594-1494cm^-1^[29,32]. The vibrational modes between 1488–1433 cm^-1^ are attributed to CH_2_/CH_3_ vibrations._._ The band between 1432–1365 cm^-1^ demonstrates CO groups (ester) stretching vibrations. Besides, spectral area between 1290–1185 cm^-1^ indicates PO_2_ asymmetric [29]. The 1135–999 cm^-1^ spectral area corresponds PO_2_ symmetric [29].

**Table 1:** ATR-FTIR wavenumber with respective vibrational modes and related chemical component in submandibular glands of vehicle-, morphine- and CGP-treated mice in the presence or absence of naloxone.

Wavenumber (cm^-1^)	Vibrational modes	Chemical component
1635 (1687–1594)	υ_N_H (Amide I)	Proteins
1550 (1594–1494)	δ_N_H (Amide II)	Proteins
1450 (1488–1433)	CH_2_/CH_3_	Lipids/proteins
1400 (1432–1365)	CO groups (ester)	Proteins
1232 (1290–1185)	PO_2_ asymmetric	Phospholipids
1031 (1135–999)	PO_2_ symmetric	Glycogen

The vibrational mode assignments were obtained from references [29–33].

### Cyclo-Gly-Pro (CGP) and opioid receptor (OR) structure assembly and interaction

The human Opioid Receptor protein FASTA sequence (Homo sapiens, access number in Gene Bank: AAA73958.1) was submitted online in I-TASSER server to predict and generate high-quality 3D predictions of this protein. The best model was verified using RAMPAGE: Assessment of the Ramachandran Plot, and Verify3D web tools to determine the spatial coherence and compatibility of the atomic model (3D) with its own amino acid sequence (1D). The CGP 3D structure was obtained from Pubchem (PubChem CID: 193540). After that, *in silico* analyses were performed to predict the interaction of both structures. AutoDOCK Vina [34] was used do predict the molecular docking using the Root-mean-square deviation of atomic positions (RMSd) and free energy calculations. PyMOL Molecular Graphics System, Version 2.0 Schrödinger, LLC, was used to visualize the CGP-OP interactions and export image files.

### Statistical analysis

In this study, data and statistical analysis comply with the recommendations on experimental design and analysis in pharmacology [35]. Values are presented as mean ± SEM. The heat map and analyses were performed using GraphPad Prism 5.0 (GraphPad Software, San Diego, CA, USA). Kolmorogov-Smirnov test was used to determine the normality of the sample distributions. Comparisons of results were performed by ANOVA, followed by Turkey’s or Dunnett’s post-test at P value <0.05.

## Results

### Effects of opioid receptors (OR) in salivary secretion and salivary composition

**Fig 2:**
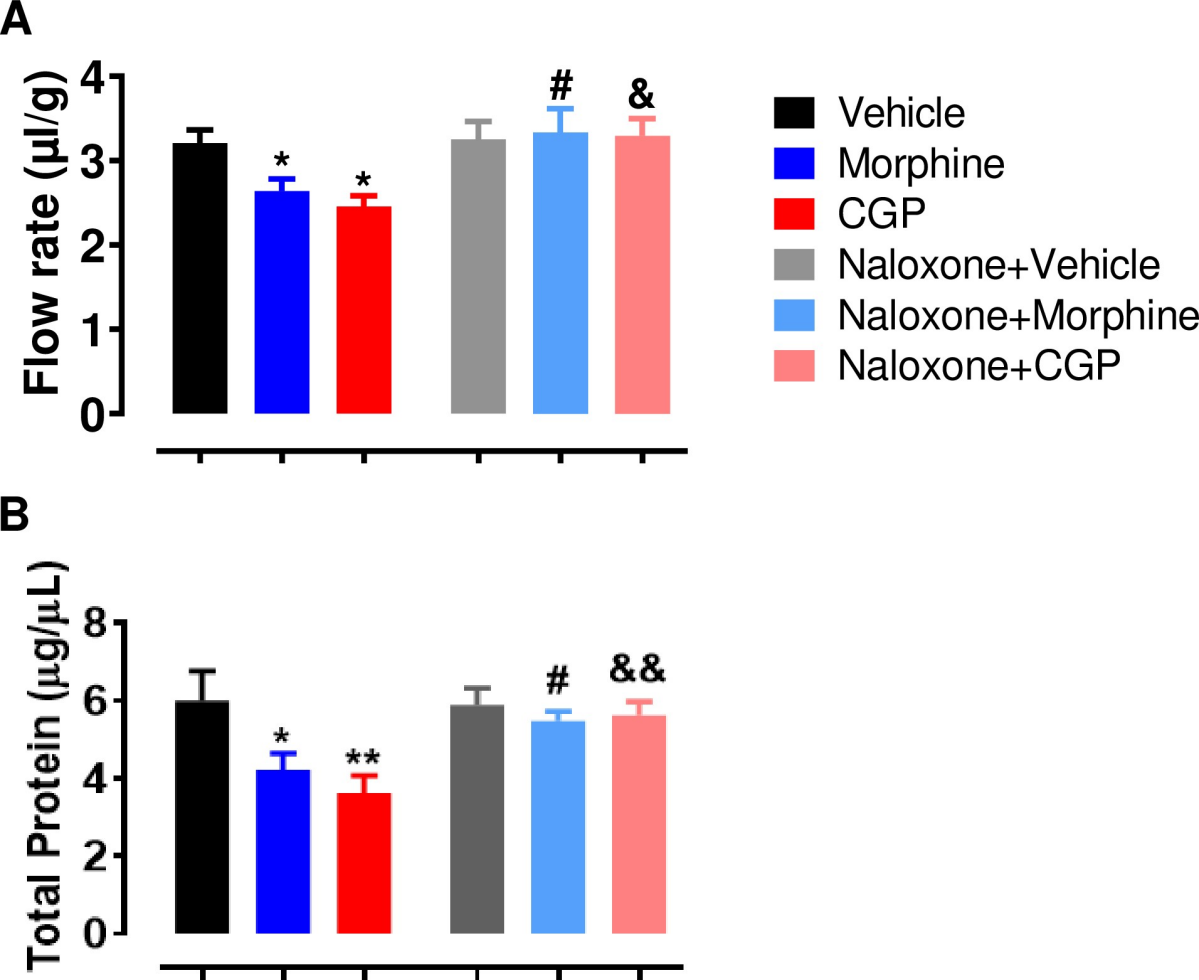
Salivary flow of Swiss mice and salivary total protein concentration after acute treatment with vehicle, morphine or CGP in the presence or absence of naloxone. CGP, Cyclo-Gly-Pro. Naloxone (15.3 μmol kg^-1^, i.p.), an opioid receptor antagonist, was administered 15 minutes before treatment with vehicle, morphine or CGP. One hour after treatment, parasympathetic stimulation was performed for salivary secretion through pilocarpine injection (2 mg kg^-1^, i.p.) and total saliva was collected for 10 minutes from the oral cavity. (A) Salivary flow was expressed as salivary secretion per gram of glandular tissue (μl/g tissue), equivalent to the ratio of the volume of salivary secretion and the weight sum of glandular parotid and submandibular. (B) Salivary total protein concentration was expressed as μg/μL. Results are represented as mean ± SEM of 6–9 animals; *P<0.05 vs. vehicle; #*P*<0.05 vs. morphine; &*P*<0.05 vs CGP. One-way ANOVA, Turkey as post hoc test.

Salivary secretion was significantly reduced in morphine compared to vehicle (~15%; *p* <0.05). Similarly, CGP significantly decreased salivary secretion compared to vehicle (~20%, *p* <0.05). Differences in salivary secretion were not significant between morphine- and CGP-treated mice (Fig 2A). To confirm the effects of the opioid receptors, naloxone was administered before the treatment with vehicle, morphine or CGP. The pilocarpine-stimulated salivary secretion remained unchanged after naloxone administration in vehicle mice (*p* >0.05; Fig 2A). On the other hand, naloxone significantly increased the salivary secretion in mice treated with CGP and morphine (~20% and 30%, respectively; *p* <0.01) (Fig 2A).

In order to analyze the effects of CGP and morphine, total protein concentration in saliva was measure using Bradford assay. Total protein concentration in saliva significantly decreased (*p*<0.01) when CGP (~45%) and morphine (~35%) was administrated and compared to vehicle (Fig 2B). Total protein concentration in saliva was similar (*p*>0.05) in morphine and CGP-treated mice. The administration of naloxone in vehicle mice kept the total protein concentration stable (*p* >0.05; Fig 2B). On the other hand, naloxone significantly increased the total protein concentration in saliva of mice treated with CGP and morphine (~40%; *p* <0.001; Fig 2B).

Dispersive X-ray analysis was performed to analyze the CGP and morphine effects on the ionic composition in saliva. The composition of potassium, chloride, sodium and sulfur ions in saliva remained unchanged after acute treatment with morphine or CGP compared to vehicle (S1 Fig).

### Maintenance of salivary glands weight under opioid receptors (OR) blockade

In order to investigate the CGP or morphine effects in salivary glands, the parotid and submandibular glands were properly weighed. The results demonstrated that the glands weight remained unchanged after acute treatment with morphine or CGP (Table 2). As expected, the salivary glands weight also did not change with the naloxone pre-treatment (Table 2).

**Table 2:** Parotid and submandibular weights from vehicle-, morphine- or CGP-treated mice in the presence or absence of naloxone.

Treatment	Submandibular weight (mg)	Parotid weight (mg)
Vehicle	51,21 ± 2,81 (9)	28,42 ± 1,55 (9)
Morphine	50,50 ± 3,05 (10)	34,44 ± 4,35 (10)
CGP	49,01 ± 2,62 (10)	31,56 ± 3,11 (10)
Naloxone+ Vehicle	50,20 ± 3,44 (6)	30,03 ± 5,38 (6)
Naloxone+Morphine	50,98 ± 4,12 (6)	32,77 ± 5,69 (6)
Naloxone+CGP	52,67 ± 5,39 (6)	31,22 ± 3,80 (6)

CGP, cyclo-Gly-Pro. *P*>0.05 vs. vehicle. One-way ANOVA, Student-Newman-Keuls as post hoc test.

### Histological changes of submandibular gland under opioid receptors (OR) blockade

To determine whether CGP promoted morphological and structural changes in salivary gland compared to the morphine treatment, we stained the submandibular sections with hematoxylin-eosin. The acinar cells, ductal cells and connective tissue remained unaltered (S2A Fig). However, submandibular glands histological analysis showed of evacuated spaces increased between serous and mucous acini in glandular parenchyma after one-hour treatment with morphine or CGP compared to the vehicle (S2B and S2C Fig).

### Effects of opioid receptors (OR) blockade on lactate levels of submandibular glands

**Fig 3:**
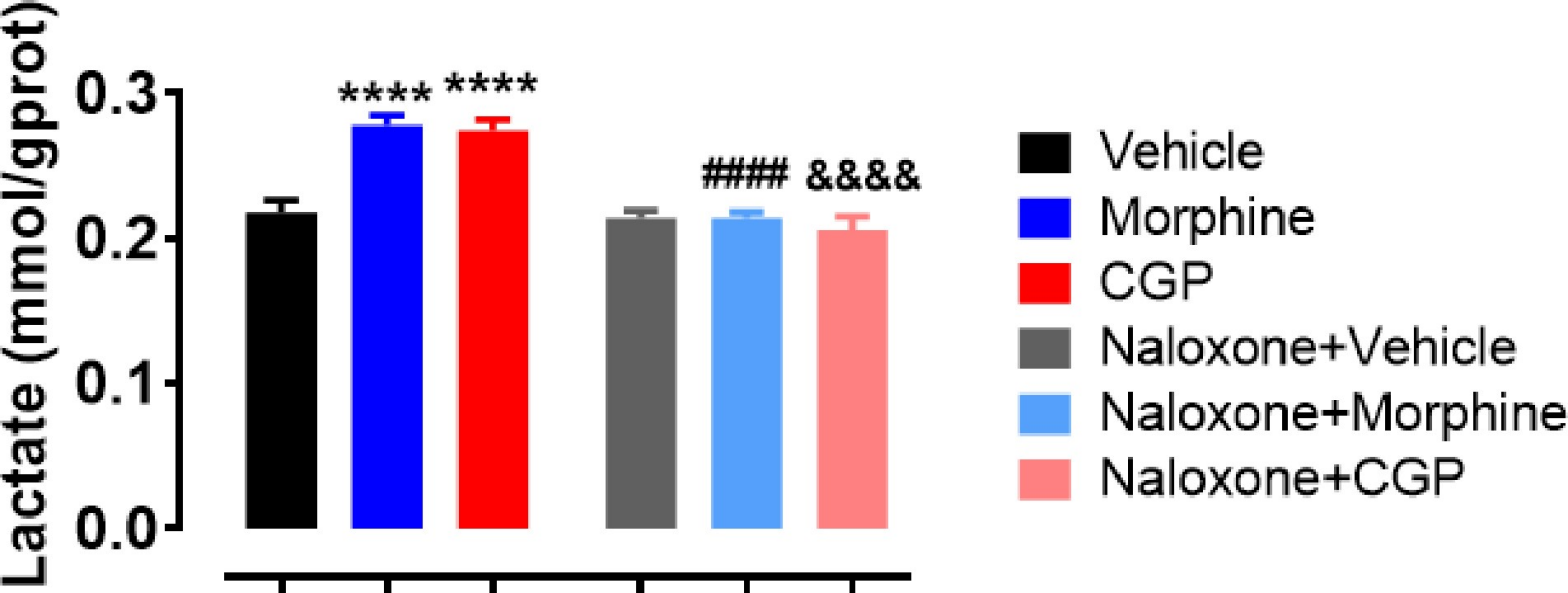
Lactate levels in submandibular gland of Swiss mice after acute treatment with vehicle, morphine or CGP in the presence or absence of naloxone. Morphine and CGP increased lactate levels in submandibular gland, but naloxone restored this parameter to the baseline. CGP, Cyclo-Gly-Pro. Results are represented as mean ± SEM of 6 animals; *P<0.05 vs. vehicle. One-way ANOVA, Dunnett as post hoc test.

Morphine (~15%) and CGP (~15%) increased (*p*<0.001) lactate levels in submandibular gland compared to vehicle (Fig 3). Lactate levels in submandibular glands were similar (*p*>0.05) in morphine and CGP-treated mice. The lactate levels in submandibular gland remained unchanged after naloxone administration in vehicle mice (*p* >0.05; Fig 3). On the other hand, naloxone significantly decreased this parameter in mice treated with CGP and morphine (~20%; *p* <0.001) (Fig 3).

### Effects of opioid receptors (OR) blockade on acetylcholinesterase activity in of submandibular glands

The acetylcholinesterase activity in submandibular gland was unaffected after acute treatment with morphine (0.16 UA/μg ± 0.02, *p* >0.05) or CGP (0.15 UA/μg ± 0.02, *p* >0.05) compared to vehicle (0.15 UA/μg ± 0.03). Besides, acetylcholinesterase activity in submandibular gland was similar (*p*>0.05) in morphine and CGP-treated mice (S3 Fig).

### Effects of opioid receptors (OR) blockade on chemical profile in submandibular by ATR-FTIR spectroscopy

**Fig 4:**
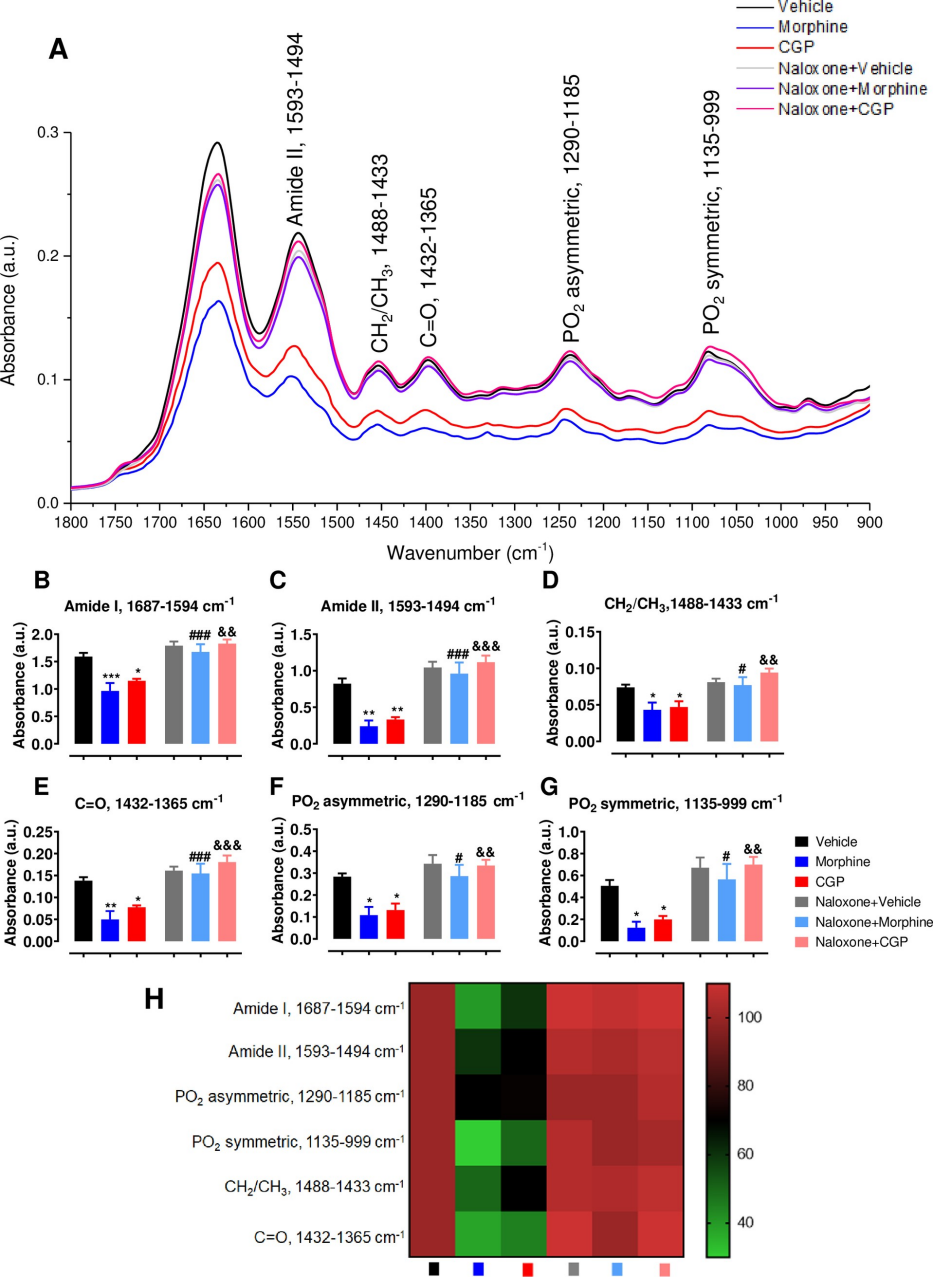
Chemical compounds profile in submandibular gland represented by means of ATR-FTIR spectra of Swiss mice after acute treatment with vehicle, morphine or CGP in the presence or absence of naloxone. (A) ATR-FTIR spectra displayed in 1800–800 cm^-1^ region. The major vibrational modes are represented, indicating changes under the acute treatment with CGP or morphine on the various chemical compounds present in the submandibular gland. These changes were reversed by naloxone pre-treatment. (B) Amide I (1687–1594 cm-1), (C) Amide II (1594–1494 cm^-1^), (D) CH2/CH3 (1488–1433 cm^-1^), (E) C = O (1432–1365 cm^-1^), (F) PO_2_ asymmetric (1290–1185 cm^-1^) and (G) PO_2_ asymmetric (1135–999 cm^-1^). (H) Heat map with the relative expression of each vibrational mode (Vehicle expression was set as 100%). Results are represented as mean ± SEM of 6 animals.

The submandibular gland infrared spectrum is a superposition of several compounds and the absorption bands in ATR-FTIR spectrum intensities are directly proportional to the components concentration. The submandibular gland spectra of vehicle-, CGP- or morphine-treated animals in presence or absence of naloxone are represented in Fig 4A.

To understand better the response of proteins, phospholipids and lipids in submandibular gland under opioid receptors (OR) blockade, we analyzed the area of molecular components using ATR-FTIR spectroscopy. There are six major vibrational modes highlighted at 1635 cm^-1^ (1687–1594 cm^-1^), 1550 cm^-1^ (1594–1494 cm^-1^), 1450 cm^-1^ (1488–1433 cm^-1^), 1400 cm^-1^ (1432–1365 cm^-1^), 1232 cm^-1^ (1290–1185 cm^-1^) and 1031 cm^-1^ (1135–999 cm^-1^), confirming the presence of proteins, lipids, phospholipids and glycogen, according to the details showed for each peak, corresponding to the specific vibration molecules (Fig 4A). The vibrational modes between 1687–1594 cm^-1^ and 1594–1494 cm^-1^, representing amide I and amide II, respectively, were reduced in morphine- or CGP-treated mice. These changes were reversed by the pre-treatment with naloxone (Fig 4B and 4C). The vibrational modes between 1488–1433 cm^-1^ and 1432–1365 cm^-1^, representing CH_2_/CH_3_ and C = O, respectively, were also reduced in morphine- or CGP-treated mice. These changes were also reversed by the pre-treatment with naloxone (Fig 4D and 4E). Besides, two vibrational modes at 1290–1185 and 1135–999 cm^-1^ were also reduced in morphine- or CGP-treated mice compare to the vehicle. These vibrational modes represent PO_2_ asymmetric and PO_2_ symmetric, respectively, and the naloxone pre-treatment reversed the changes in both submandibular glands components (Fig 4F and 4G). A heat map with the mean relative changes clearly demonstrates the expression in these vibrational modes (Fig 4H).

### Assembly and interaction of the OR and CGP structure

**Fig 5:**
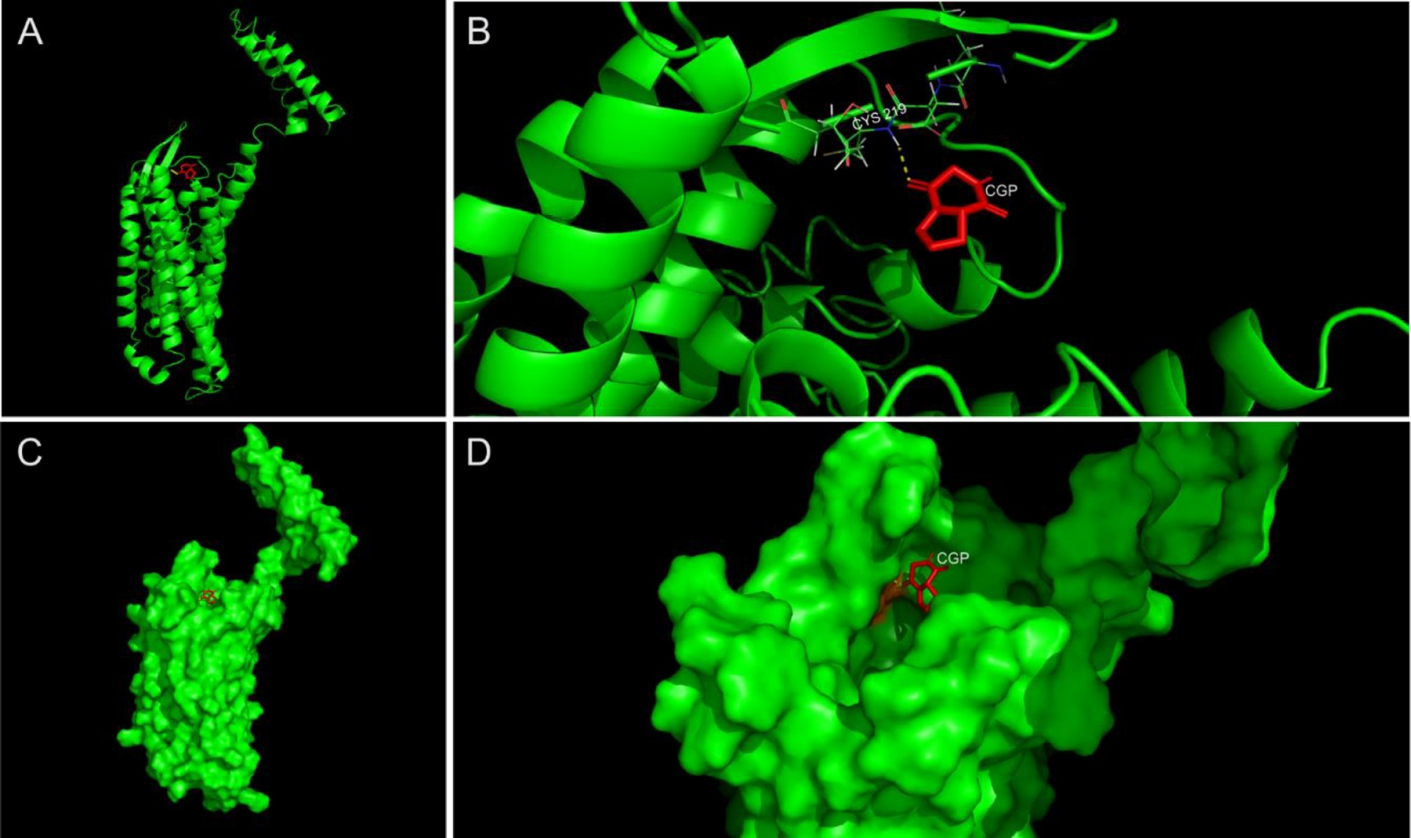
Assembly and interaction of the OR and CGP structure. (A) Full cartoon structure of OP (green) coupled with CGP (red). (B) Expanded image of binding site from docking analysis. Polar contacts are shown by yellow dashes between the CGP (red) and the OR Cys219 residue. (C) Full surface structure of OP (green) coupled with CGP (red). (D) OP framework and conformational interaction site (green). The binding spot to CGP (red) is presented in orange.

*In silico* modeling of OR and molecular docking of OR and CGP were performed by I-TASSER server. Fig 5A shows the full cartoon structure of OP (green) interacting with CGP (red). The extended view of the interaction site from docking analysis demonstrated the polar contacts (yellow dashes) between the CGP (red) and the OR Cys219 residue (Fig 5B). Fig 5C shows the full surface of the structure of OP (green) coupled with CGP (red) and Fig 5D presents the OP framework and conformational interaction site.

## Discussion

The opioid agonist depressant mechanisms on salivary secretion and salivary glandular tissue effects remain unclear. Additionally, the potential effects of therapeutic agents in secondary target organs as salivary glands still need to be carefully investigated. Given the ongoing attempts to describe opioid side effects on salivary function, it is clearly important to understand and characterize the CGP effects in saliva and salivary glands composition compared with morphine. We showed that either CGP or morphine promoted the reduction in flow rates and in salivary protein concentration, increased lactate levels in glandular tissue and resulted in severe changes in chemical components in submandibular glands. Conversely, the naloxone (a non-selective opioid receptor antagonist) reversed this alteration in saliva and submandibular glands.

Human salivary secretion was decreased from 1 to 4 hours after administration of morphine intravenously [36]. Besides, the treatment with morphine (6 mg/kg) also inhibited the salivary flow in rats [14]. As expect, in this present study the acute treatment with morphine promoted salivary secretion reduction. Furthermore, for the first time, we showed that CGP also reduces salivary secretion in mice.

Previous studies have demonstrated that naloxone partially restores pilocarpine-stimulated and parasympathetic nerve stimulated-salivary secretion under morphine treatment [14,19]. As expected, our data corroborates previous studies demonstrating that naloxone reversed the morphine depressant effects in salivary flow. We further investigated whether the salivary flow changes elicited by CGP were also reversed by naloxone, due to its nonspecific antagonistic action on mu, kappa and delta opioid receptors [37]. It is important to point out that the opioid receptors blockage by naloxone was also able to reverse the inhibitory effect caused by CGP and to restore the salivary flow, indicating the CGP effect in opioid receptors as described to morphine treatment. So, these data suggest similar side effects on salivary glands by CGP or morphine.

Salivary ionic composition remained unchanged after acute treatment with CGP or morphine (5 mg kg^-1^). In previous research, morphine at a dose of 25 mg kg^-1^did not alter the sodium and calcium concentrations in parotid saliva of rats; on the other hand, led to an increase in salivary potassium concentration [19]. Interestingly, morphine at a dose of 6 mg kg^-1^ did not alter the presence of salivary potassium concentration, however it was able to reduce the salivary calcium and increase salivary sodium concentration [14]. It is possible that samples collected using different methods contributed to these different results. Despite the contradictory reports evaluating morphine effects on ions composition under lower and higher doses, our data on CGP showing no changes in salivary ionic composition has never been reported and suggests that CGP may not be involved in the regulation of several channels that regulate ionic composition in salivary glands.

We have also shown that CGP, as well as morphine, decreased salivary protein concentration in mice, which is corroborated elsewhere by the demonstration that an acute treatment with morphine (6 mg kg^-1^) decreased protein concentration in saliva from the rat submandibular gland [14]. Considering that sympathetic activity on salivary glands is the most important control of salivary protein secretion and pointing out that the presence of opioid receptors in submandibular and parotid glands was never demonstrated, the CGP or morphine effects to reduce protein concentration in saliva could be due a direct interference of cAMP in cells that express opioid receptors. Moreover, the tolerance to opioid receptors changes the pathway signal transduction by the cAMP-dependent protein kinase [38,39]. Thus, if opioid receptors are expressed in salivary glands, probably morphine and CGP reduces the cAMP directly in these glands, which is a key mechanism that may be involved in reduced protein secretion in saliva. However, another explanation for morphine and CGP inhibitory effects on salivary protein concentration may be due to the opioid receptors presence that might have inhibited preganglionic or ganglionic sympathetic nerve projecting to salivary gland [40–42]. In both hypotheses, it seems clear that our study indicates inhibition of effects promoted by CGP and morphine by interaction with opioid receptors.

The reduction in salivary protein concentration and in Amide I/Amide II of submandibular promoted by opioid agonists is a characteristic of tissues that have low sympathetic activity and/or low glucose utilization, likely because they need to have a low energy status [43]. Besides, we also showed that both treatments are able to reduce glycogen, indicating influence of opioid system in glycogen metabolism on submandibular glands [44]. Specifically, the present study describes evacuated spaces (previously occupied by secretory acini) between acinar and ductal cells in glandular parenchyma of submandibular glands under morphine- and CGP-treatment. The fast (1h) effect of morphine and CGP suggests that it is not solely a consequence of reduction of parasympathetic/sympathetic activity. This is in agreement with morphine-induced apoptosis of murine J774 cells mediated through TGF-beta [45]. Notably, this change is reinforced in morphine-treated mice. Considering the acute morphological changes with morphine and CGP, we can still propose induction of more profound morphological changes during chronic treatment. However, these effects could be promoted by the activation of kappa-, delta-, and/or mu-opioid-receptor by morphine and CGP in submandibular ganglion [18,24,46].

The CH_2_ reduction in lipids [47] after morphine and CGP treatments suggests a decrease in lipid rafts that translocate proteins from intracellular structures to the plasma membrane [48], as well as decreasing energy status. Opioid agonists reduced PO_2_ asymmetric of phospholipids, indicating damage in plasma membrane, which is also an apoptotic characteristic [49].

Considering the similar changes in salivary secretion and submandibular composition promoted by both therapeutic agents, we can consider that CGP have similar inhibitory effect on autonomic activity to salivary glands as described to morphine [18]. The CGP antinociceptive effect was antagonized by naloxone, a non-selective opioid receptor antagonist, suggesting that CGP effect may also occur through opioid receptor at the supraspinal level [24,50]. CGP increased neuronal activity in midbrain periaqueductal gray (PAG), a key relay station in the processing of nociceptive information in central nervous system [24,51] and that is interconnected with the hypothalamus [52]. Opioid receptors are spread in the hypothalamus [53] and these hypothalamic neurons may have inhibitory projections to superior salivary nucleus, from where ganglionic fibers of parasympathetic nervous system spread to submandibular glands [54–56]. Morphine also activates PAG [57], which suggest similar repercussion of CGP.

**Fig 6:**
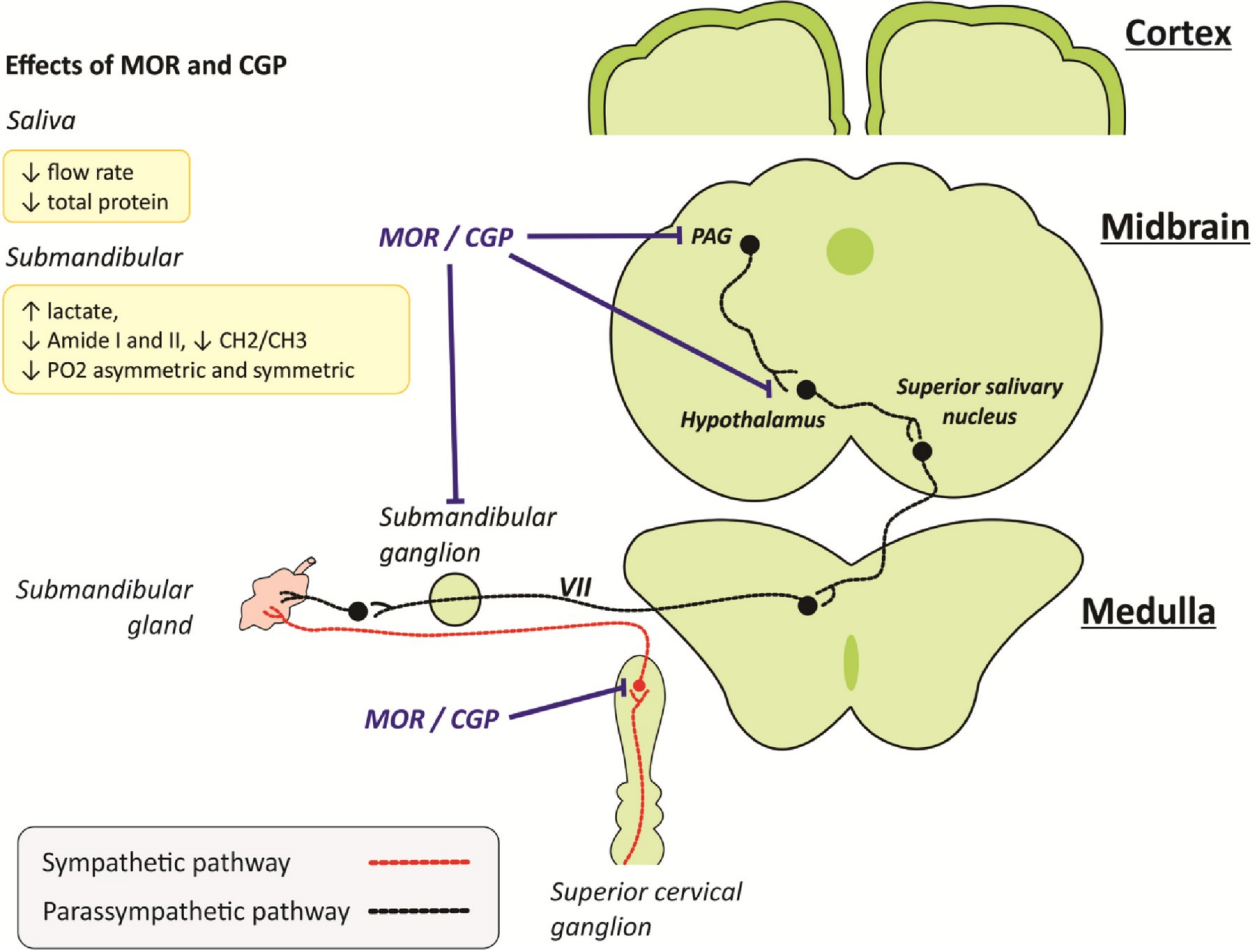
Schematic draw of a proposed pathway for morphine- and CGP-induced regulation of midbrain and autonomic ganglions. Arrows indicate the salivary and submandibular gland contents regulations (↑increase, ↓decrease) under morphine or CGP treatments. Blue line indicates the inhibitory effect of morphine and CGP to autonomic pathway to submandibular glands. Red and black lines indicate the sympathetic and parasympathetic pathway to submandibular gland. Opioid receptors are expressed in PAG [51], hypothalamus [52,53], submandibular ganglia [18,46] and superior cervical [41,42] ganglia. CGP, cyclo-Gly-Pro.

The muscarinic receptors sensitivity s in salivary glands was not reduced by morphine [19]. Therefore, we evaluated whether the salivary secretion reduction after treatment with CGP and morphine could be explained by acetylcholine decreased levels in the extracellular fluid, which could be demonstrated by acetylcholinesterase enzyme increased activity. Previous studies have shown an increase in brain acetylcholinesterase expression, 30 minutes after morphine injection (10 mg kg^-1^of morphine), indicating a higher enzyme activity and further acetylcholine degradation.[58]. However, another study showed that morphine chronic administration decreases acetylcholinesterase activity in the midbrain [59]. It is noteworthy that the acute effect of morphine and CGP on acetylcholinesterase activity in salivary gland has never been reported. Bearing in mind that the expected reduction of acetylcholine in synaptic cleft due to lower parasympathetic activity [18] is associated with similar acetylcholinesterase activity in submandibular glands after morphine and CGP treatment, it is expected that the acetylcholine presence in synaptic cleft can be further reduced due to the acetylcholine/acetylcholinesterase ratio. Furthermore, these data emphasize that the reduction of pilocarpine–induced salivary secretion by morphine and CGP is likely to be prejunctional. Considering the present results and previous reports, we suggest a central and autonomic-pathway leading to changes in submandibular gland and hyposalivation by morphine and CGP (Fig 6).

To the best of our knowledge, this is the first report that demonstrates the salivary hyposecretion elicited by CGP, which is mediated by the opioid receptors’ system and such effect was reversed by naloxone. The present study also provides new evidence for an inhibitory morphine effect in pilocarpine-salivary secretion mediated by the opioid system in mice. Morphine and CGP also reduced salivary protein concentration and increased the lactate in submandibular gland and naloxone reverted both alterations. Morphine and CGP also reduced several infrared vibrational modes representing Amide I, Amide II, CH_2_/CH_3_, C = O, PO_2_ asymmetric and PO_2_ symmetric, which was blocked by pre-treatment with naloxone. To confirm the pathway to CGP effects, the *in silico* docking analysis demonstrate the polar contacts interaction between the CGP and opioid receptor Cys219 residue. Altogether, we showed that salivary hypofunction and glandular changes elicited by CGP may occur through opioid receptor suggesting that opioid receptors blockage in superior cervical ganglion and submandibular ganglion may be a possible strategy to restore salivary secretion while maintaining antinociceptive effects due its effects in central nervous system.

## Supporting information

S1 Fig Effect of acute treatment with CGP and morphine on ionic composition present in stimulated saliva. (A-D) Inorganic elements of pilocarpine-stimulated saliva were evaluated the by fluorescent X-ray method. Concentrations of potassium (A), sulfur (B), chloride (C) and sodium (D) ions in the stimulated saliva remained unchanged after acute treatment with CGP and morphine. CGP, cyclo-Gly-Pro. Results are mean ± SEM of 6 animals; *P*>0.05 vs. vehicle. One-way ANOVA, Dunnett as post hoc test.
(PPT)

S2 Fig Photomicrograph of submandibular gland from Swiss mice after 1h-treatment with vehicle, morphine and CGP. (A) The submandibular gland morphology is intact in vehicle-treated mice. (B) The acute treatment with morphine was able to increase the evacuated spaces (arrows) between the mucosal and serous acini in the submandibular gland parenchyma. (C) This change was not markedly evident after one hour of CGP treatment. Images are representative of 8 animals in each group. CGP, cyclo-Gly-Pro. Magnification, x400; scale bar, 20 μm.
(PPT)

S3 Fig Activity of AChE enzyme in submandibular glands from Swiss mice after acute treatment with vehicle, morphine and CGP. CGP, cyclo-Gly-Pro. Results are mean ± SEM of 5 animals; *p* >0.05 vs. vehicle. One-way ANOVA, Dunnett as post hoc test.
(PPT)

## References

[1] Humphrey SP, Williamson RT. A review of saliva: Normal composition, flow, and function. J Prosthet Dent. 2001;85: 162–169. 10.1067/mpr.2001.113778 11208206

[2] Aps JKM, Martens LC. Review: The physiology of saliva and transfer of drugs into saliva. Forensic Sci Int. 2005;150: 119–131. 10.1016/j.forsciint.2004.10.026 15944052

[3] Ruhl S. The scientific exploration of saliva in the post-proteomic era: from database back to basic function. Expert Rev Proteomics. 2012;9: 85–96. 10.1586/epr.11.80 22292826 PMC3289089

[4] Emmelin N. Nerve interactions in salivary glands. J Dent Res. 1987;66: 509–517. 10.1177/00220345870660022101 3305629

[5] Sabino-Silva R, Ceroni A, Koganezawa T, Michelini LC, Machado UF, Antunes VR. Baroreceptor-mediated activation of sympathetic nerve activity to salivary glands. Physiol Behav. 2012;107: 390–6. 10.1016/j.physbeh.2012.09.012 23022472

[6] Proctor GB, Carpenter GH. Regulation of salivary gland function by autonomic nerves. Auton Neurosci Basic Clin. 2007;133: 3–18. 10.1016/j.autneu.2006.10.006 17157080

[7] Carpenter GH, Garrett JR, Hartley RH, Proctor GB. The influence of nerves on the secretion of immunoglobulin A into submandibular saliva in rats. J Physiol. 1998;512 (Pt 2: 567–73. 10.1111/j.1469-7793.1998.567be.x 9763644 PMC2231220

[8] Garrett JR. The proper role of nerves in salivary secretion: a review. J Dent Res. 1987;66: 387–397. 10.1177/00220345870660020201 3305622

[9] Sabino-Silva R, Alves-Wagner ABT, Burgi K, Okamoto MM, Alves AS, Lima G a, et al SGLT1 protein expression in plasma membrane of acinar cells correlates with the sympathetic outflow to salivary glands in diabetic and hypertensive rats. Am J Physiol Endocrinol Metab. 2010;299: E1028–37. 10.1152/ajpendo.00395.2010 20841505

[10] Renzi A, Colombari E, Mattos Filho TR, Silveira JE, Saad WA, Camargo LA, et al Involvement of the central nervous system in the salivary secretion induced by pilocarpine in rats. J Dent Res. 1993;72: 1481–4. 10.1177/00220345930720110401 8227698

[11] Meert TF, Vermeirsch H a. A preclinical comparison between different opioids: Antinociceptive versus adverse effects. Pharmacol Biochem Behav. 2005;80: 309–326. 10.1016/j.pbb.2004.12.002 15680184

[12] Rougeot C, Robert F, Menz L, Bisson J-F, Messaoudi M. Systemically active human opiorphin is a potent yet non-addictive analgesic without drug tolerance effects. J Physiol Pharmacol. 2010;61: 483–90. Available: http://www.ncbi.nlm.nih.gov/pubmed/20814077 20814077

[13] COSMO A. DiFAZIO;CHEN PIN-YANG. The Influence of Morphine on Excess Lactate Production. Anesth Analg. 1971;50: 211–216. Available: https://insights.ovid.com/pubmed?pmid=5102636 5102636

[14] Abdollahi M, Safarhamidi H. Protection by nitric oxide of morphine-induced inhibition of rat submandibular gland function. Pharmacol Res. 2002;45: 87–92. 10.1006/phrs.2001.0910 11846618

[15] Hashemi N, Mohammadirad A, Bayrami Z, Khorasani R, Vosough S, Aliahmadi A, et al Restoration of morphine-induced alterations in rat submandibular gland function by N-methyl-D-aspartate agonist. Acta Biol Hung. 2006;57: 283–294. 10.1556/ABiol.57.2006.3.2 17048692

[16] Crosara KTB, Zuanazzi D, Moffa EB, Xiao Y, Machado MA de AM, Siqueira WL. Revealing the Amylase Interactome in Whole Saliva Using Proteomic Approaches. Biomed Res Int. Hindawi; 2018;2018: 1–15. 10.1155/2018/6346954 PMC5831883 29662892

[17] Miwa Y, Saeki M, Yamaji A, Maeda S, Saito K. Effect of morphine on secretion of amylase from isolated parotid acini. Life Sci. 1996;59: 1809–19. Available: http://www.ncbi.nlm.nih.gov/pubmed/8937508 doi: 10.1016/0024-3205(96)00524-3 8937508 10.1016/0024-3205(96)00524-3

[18] Endoh T, Suzuki T. The regulating manner of opioid receptors on distinct types of calcium channels in hamster submandibular ganglion cells. Arch Oral Biol. 1998;43: 221–33. Available: http://www.ncbi.nlm.nih.gov/pubmed/9631175 doi: 10.1016/s0003-9969(98)00002-8 9631175 10.1016/s0003-9969(98)00002-8

[19] Bowen SR, Carpenter FG. Morphine depression and tolerance of nerve-induced parotid secretion. Br J Pharmacol. 1979;65: 7–13. Available: http://www.ncbi.nlm.nih.gov/pubmed/760892 doi: 10.1111/j.1476-5381.1979.tb17327.x 760892 10.1111/j.1476-5381.1979.tb17327.x PMC1668469

[20] Prasad C. Bioactive cyclic dipeptides. Peptides. 1995;16: 151–64. Available: http://www.ncbi.nlm.nih.gov/pubmed/7716068 doi: 10.1016/0196-9781(94)00017-z 7716068 10.1016/0196-9781(94)00017-z

[21] Burgos-Ramos E, Martos-Moreno G a, López MG, Herranz R, Aguado-Llera D, Egea J, et al The N-terminal tripeptide of insulin-like growth factor-I protects against beta-amyloid-induced somatostatin depletion by calcium and glycogen synthase kinase 3 beta modulation. J Neurochem. 2009;109: 360–370. doi:JNC5980 [pii]\r10.1111/j.1471-4159.2009.05980.x 10.1111/j.1471-4159.2009.05980.x 19220704

[22] Guan J, Gluckman PD. IGF-1 derived small neuropeptides and analogues: A novel strategy for the development of pharmaceuticals for neurological conditions. Br J Pharmacol. 2009;157: 881–891. 10.1111/j.1476-5381.2009.00256.x 19438508 PMC2737647

[23] Hammond DL, Washington JD. Antagonism of L-baclofen-induced antinociception by CGP 35348 in the spinal cord of the rat. Eur J Pharmacol. 1993;234: 255–62. Available: http://www.ncbi.nlm.nih.gov/pubmed/8387011 doi: 10.1016/0014-2999(93)90961-g 8387011 10.1016/0014-2999(93)90961-g

[24] Ferro JN de S, de Aquino FLT, de Brito RG, Dos Santos PL, Quintans J de SS, de Souza LC, et al Cyclo-Gly-Pro, a cyclic dipeptide, attenuates nociceptive behaviour and inflammatory response in mice. Clin Exp Pharmacol Physiol. 2015;42: 1287–95. 10.1111/1440-1681.12480 26277051

[25] Charan J, Kantharia N. How to calculate sample size in animal studies? J Pharmacol Pharmacother. 2013;4: 303 10.4103/0976-500X.119726 24250214 PMC3826013

[26] Zoukhri D, Kublin CL. Impaired Neurotransmitter Release from Lacrimal and Salivary Gland Nerves of a Murine Model of Sjogren’s Syndrome. Invest Ophthalmol Vis Sci. 2001;42: 925–932. 10.1016/j.biotechadv.2011.08.021.Secreted 11274068 PMC3241007

[27] Sabino-Silva R, Okamoto MM, David-Silva A, Mori RC, Freitas HS, Machado UF. Increased SGLT1 expression in salivary gland ductal cells correlates with hyposalivation in diabetic and hypertensive rats. Diabetol Metab Syndr. 2013;5: 64 10.1186/1758-5996-5-64 24499577 PMC4029169

[28] Bradford MM. A rapid and sensitive method for the quantitation of microgram quantities of protein utilizing the principle of protein-dye binding. Anal Biochem. 1976;72: 248–54. Available: http://www.ncbi.nlm.nih.gov/pubmed/942051 doi: 10.1006/abio.1976.9999 942051 10.1016/0003-2697(76)90527-3

[29] Khaustova S, Shkurnikov M, Tonevitsky E, Artyushenko V, Tonevitsky A. Noninvasive biochemical monitoring of physiological stress by Fourier transform infrared saliva spectroscopy. Analyst. 2010;135: 3183–92. 10.1039/c0an00529k 20953513

[30] Schultz CP, Ahmed MK, Dawes C, Mantsch HH. Thiocyanate levels in human saliva: quantitation by Fourier transform infrared spectroscopy. Anal Biochem. 1996;240: 7–12. 10.1006/abio.1996.0323 8811872

[31] Christersson CE, Lindh L, Arnebrant T. Film-forming properties and viscosities of saliva substitutes and human whole saliva. Eur J Oral Sci. 2000;108: 418–25. Available: http://www.ncbi.nlm.nih.gov/pubmed/11037758 doi: 10.1034/j.1600-0722.2000.108005418.x 11037758 10.1034/j.1600-0722.2000.108005418.x

[32] Júnior PCC, Strixino JF, Raniero L. Analysis of saliva by Fourier transform infrared spectroscopy for diagnosis of physiological stress in athletes. Res Biomed Eng. 2015;31: 116–124. 10.1590/2446-4740.0664

[33] Zohdi V, Whelan DR, Wood BR, Pearson JT, Bambery KR, Black MJ. Importance of Tissue Preparation Methods in FTIR Micro-Spectroscopical Analysis of Biological Tissues: ‘Traps for New Users.’ Tajmir-Riahi H-A, editor. PLoS One. Public Library of Science; 2015;10: e0116491 10.1371/journal.pone.0116491 25710811 PMC4339720

[34] Trott O, Olson AJ. AutoDock Vina: improving the speed and accuracy of docking with a new scoring function, efficient optimization, and multithreading. J Comput Chem. NIH Public Access; 2010;31: 455–61. 10.1002/jcc.21334 19499576 PMC3041641

[35] Curtis MJ, Bond RA, Spina D, Ahluwalia A, Alexander SPA, Giembycz MA, et al Experimental design and analysis and their reporting: new guidance for publication in BJP. Br J Pharmacol. 2015;172: 3461–71. 10.1111/bph.12856 26114403 PMC4507152

[36] Westerling D, Höglund P, Lundin S, Svedman P. Transdermal administration of morphine to healthy subjects. Br J Clin Pharmacol. 1994;37: 571–576. 10.1111/j.1365-2125.1994.tb04306.x 7917776 PMC1364817

[37] Peres e Serra A, Ashmawi HA. Influence of naloxone and methysergide on the analgesic effects of low-level laser in an experimental pain model. Rev Bras Anestesiol. 2010;60: 302–10. 10.1016/S0034-7094(10)70037-4 20682161

[38] Fundytus ME, Coderre TJ. Chronic inhibition of intracellular Ca2+ release or protein kinase C activation significantly reduces the development of morphine dependence. Eur J Pharmacol. 1996;300: 173–181. doi:0014299995008713 [pii] 10.1016/0014-2999(95)00871-3 8739205

[39] Smith FL, Lohmann AB, Dewey WL. Involvement of phospholipid signal transduction pathways in morphine tolerance in mice. Br J Pharmacol. 1999;128: 220–6. 10.1038/sj.bjp.0702771 10498855 PMC1571610

[40] TRENDELENBURG U. The action of morphine on the superior cervical ganglion and on the nictitating membrane of the cat. Br J Pharmacol Chemother. 1957;12: 79–85. Available: http://www.ncbi.nlm.nih.gov/pubmed/13413156 doi: 10.1111/j.1476-5381.1957.tb01366.x 13413156 10.1111/j.1476-5381.1957.tb01366.x PMC1509639

[41] Zhang C, Bachoo M, Polosa C. The receptors activated by exogenous and endogenous opioids in the superior cervical ganglion of the cat. Brain Res. 1993;622: 211–214. 10.1016/0006-8993(93)90821-4 8242358

[42] Zhang C, Bachoo M, Morales M, Collier B, Polosa C. The site of inhibitory action of endogenous opioids in the superior cervical ganglion of the cat. Brain Res. Elsevier; 1995;683: 59–64. 10.1016/0006-8993(95)00360-3 7552345

[43] Isabella R, Raffone E. Does ovary need D-chiro-inositol? J Ovarian Res. 2012;5: 14 10.1186/1757-2215-5-14 22587479 PMC3447676

[44] Gill A, Gao N, Lehrman MA. Rapid activation of glycogen phosphorylase by the endoplasmic reticulum unfolded protein response. J Biol Chem. 2002;277: 44747–53. 10.1074/jbc.M205001200 12223475

[45] Singhal PC, Kapasi AA, Franki N, Reddy K. Morphine-induced macrophage apoptosis: the role of transforming growth factor-beta. Immunology. 2000;100: 57–62. Available: http://www.ncbi.nlm.nih.gov/pubmed/10809959 doi: 10.1046/j.1365-2567.2000.00007.x 10809959 10.1046/j.1365-2567.2000.00007.x PMC2326991

[46] Endoh T, Suzuki T. The effects of delta-opioid receptor agonists on synaptic transmission in hamster submandibular ganglion. Bull Tokyo Dent Coll. 1995;36: 87–90. Available: http://www.ncbi.nlm.nih.gov/pubmed/8689748 8689748

[47] Huang N, Short M, Zhao J, Wang H, Lui H, Korbelik M, et al Full range characterization of the Raman spectra of organs in a murine model. Opt Express. 2011;19: 22892–909. Available: http://www.ncbi.nlm.nih.gov/pubmed/22109167 doi: 10.1364/OE.19.022892 22109167 10.1364/OE.19.022892

[48] Feng S, Huang S, Lin D, Chen G, Xu Y, Li Y, et al Surface-enhanced Raman spectroscopy of saliva proteins for the noninvasive differentiation of benign and malignant breast tumors. Int J Nanomedicine. 2015;10: 537–47. 10.2147/IJN.S71811 25609959 PMC4298339

[49] Tegeder I, Geisslinger G. Opioids as modulators of cell death and survival—unraveling mechanisms and revealing new indications. Pharmacol Rev. 2004;56: 351–69. 10.1124/pr.56.3.2 15317908

[50] Heinricher MM, Tavares I, Leith JL, Lumb BM. Descending control of nociception: Specificity, recruitment and plasticity. Brain Res Rev. 2009;60: 214–25. 10.1016/j.brainresrev.2008.12.009 19146877 PMC2894733

[51] Bandler R, Shipley MT. Columnar organization in the midbrain periaqueductal gray: modules for emotional expression? Trends Neurosci. 1994;17: 379–89. Available: http://www.ncbi.nlm.nih.gov/pubmed/7817403 doi: 10.1016/0166-2236(94)90047-7 7817403 10.1016/0166-2236(94)90047-7

[52] Yaksh TL, Yeung JC, Rudy TA. Systematic examination in the rat of brain sites sensitive to the direct application of morphine: observation of differential effects within the periaqueductal gray. Brain Res. 1976;114: 83–103. Available: http://www.ncbi.nlm.nih.gov/pubmed/963546 doi: 10.1016/0006-8993(76)91009-x 963546 10.1016/0006-8993(76)91009-x

[53] Yaksh TL. Spinal systems and pain processing: Development of novel analgesic drugs with mechanistically defined models. Trends Pharmacol Sci. 1999;20: 329–337. 10.1016/s0165-6147(99)01370-x 10431212

[54] Segal K, Lisnyansky I, Nageris B, Feinmesser R. Parasympathetic innervation of the salivary glands. Oper Tech Otolaryngol Neck Surg. Elsevier; 1996;7: 333–338. 10.1016/S1043-1810(96)80005-4

[55] Lin LH, Agassandian K, Fujiyama F, Kaneko T, Talman WT. Evidence for a glutamatergic input to pontine preganglionic neurons of the superior salivatory nucleus in rat. J Chem Neuroanat. 2003;25: 261–268. doi:S0891061803000334 [pii] 10.1016/s0891-0618(03)00033-4 12842271

[56] Mitoh Y, Funahashi M, Kobashi M, Matsuo R. Excitatory and inhibitory postsynaptic currents of the superior salivatory nucleus innervating the salivary glands and tongue in the rat. Brain Res. 2004;999: 62–72. 10.1016/j.brainres.2003.11.053 14746922

[57] Hao S, Liu S, Zheng X, Zheng W, Ouyang H, Mata M, et al The role of TNFα in the periaqueductal gray during naloxone-precipitated morphine withdrawal in rats. Neuropsychopharmacology. 2011;36: 664–76. 10.1038/npp.2010.197 21068718 PMC3055683

[58] Mohanakumar KP, Sood PP. Acetylcholinesterase changes in the central nervous system of mice during the development of morphine tolerance addiction and withdrawal. Brain Res Bull. 1983;10: 589–96. Available: http://www.ncbi.nlm.nih.gov/pubmed/6683583 doi: 10.1016/0361-9230(83)90026-6 6683583 10.1016/0361-9230(83)90026-6

[59] Neugebauer NM, Einstein EB, Lopez MB, McClure-Begley TD, Mineur YS, Picciotto MR. Morphine dependence and withdrawal induced changes in cholinergic signaling. Pharmacol Biochem Behav. 2013;109: 77–83. 10.1016/j.pbb.2013.04.015 23651795 PMC3690589

