# Supplementary figures and images for: Restoration of Cyclo-Gly-Pro-induced salivary hyposecretion and submandibular composition by naloxone in mice
Source: PLoS One. 2020 Mar 10;15(3):e0229761. doi: 10.1371/journal.pone.0229761 (PMC7064257; doi:10.1371/journal.pone.0229761)

## Slide 1
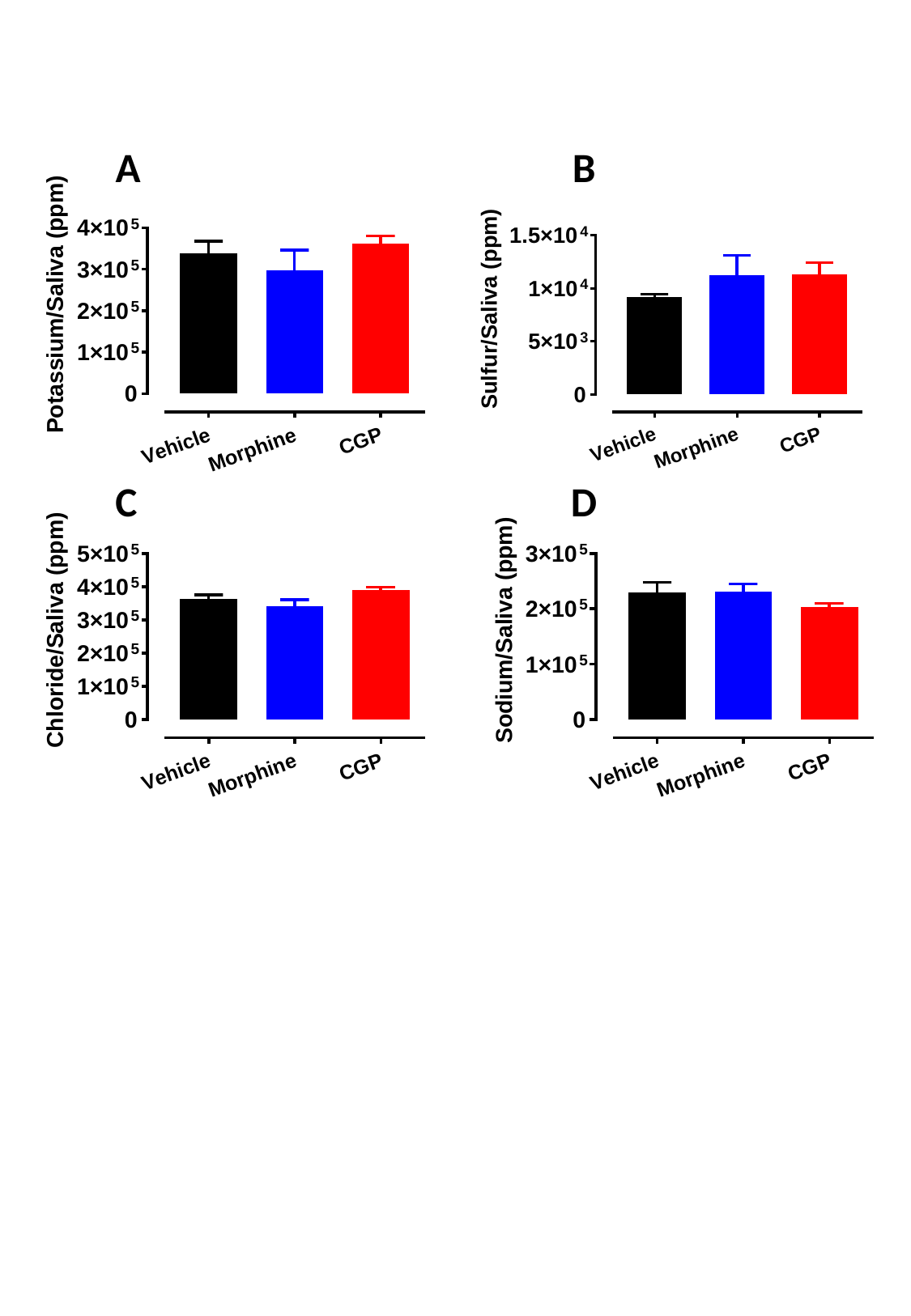

Supplement: S1 Fig — (A-D) Inorganic elements of pilocarpine-stimulated saliva were evaluated the by fluorescent X-ray method. Concentrations of potassium (A), sulfur (B), chloride (C) and sodium (D) ions in the stimulated saliva remained unchanged after acute treatment with CGP and morphine. CGP, cyclo-Gly-Pro. Results are mean ± SEM of 6 animals; P>0.05 vs. vehicle. One-way ANOVA, Dunnett as post hoc test. (PPT) [file pone.0229761.s001.ppt]

## Slide 1
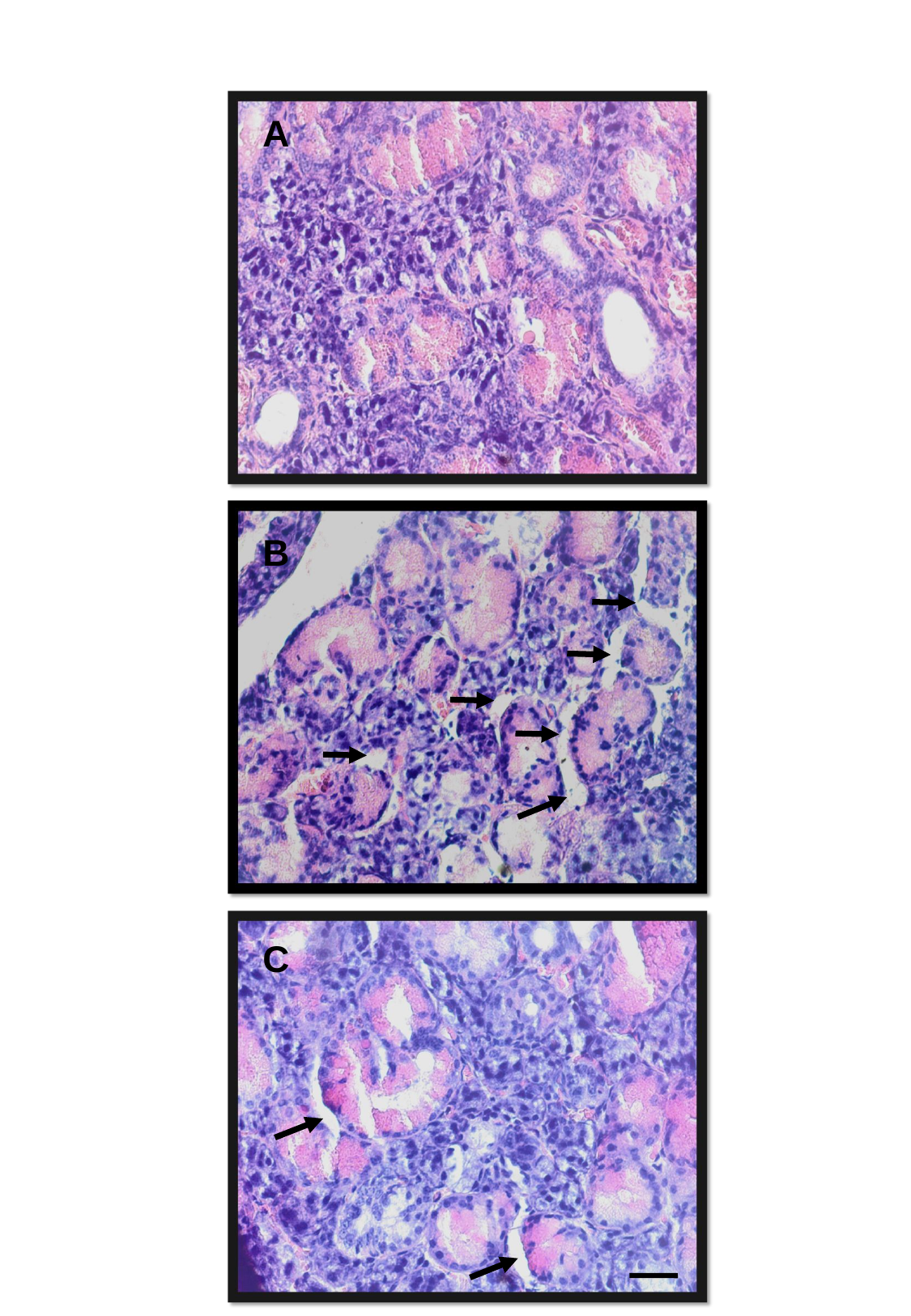

A
B
C

Supplement: S2 Fig — (A) The submandibular gland morphology is intact in vehicle-treated mice. (B) The acute treatment with morphine was able to increase the evacuated spaces (arrows) between the mucosal and serous acini in the submandibular gland parenchyma. (C) This change was not markedly evident after one hour of CGP treatment. Images are representative of 8 animals in each group. CGP, cyclo-Gly-Pro. Magnification, x400; scale bar, 20 μm. (PPT) [file pone.0229761.s002.ppt]

## Slide 1
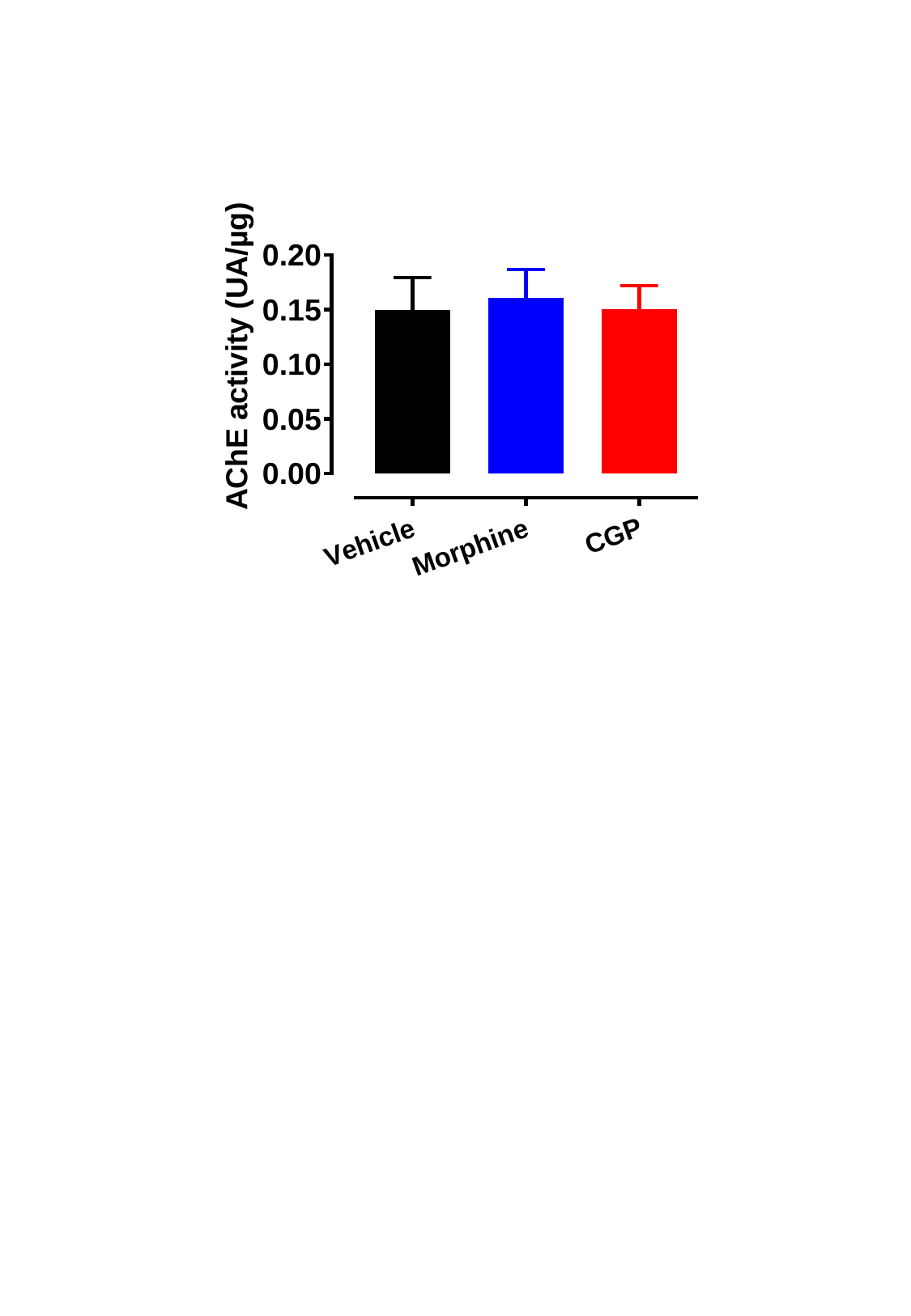

Supplement: S3 Fig — CGP, cyclo-Gly-Pro. Results are mean ± SEM of 5 animals; p >0.05 vs. vehicle. One-way ANOVA, Dunnett as post hoc test. (PPT) [file pone.0229761.s003.ppt]
